# MapCell: Learning a Comparative Cell Type Distance Metric With Siamese Neural Nets With Applications Toward Cell-Type Identification Across Experimental Datasets

**DOI:** 10.3389/fcell.2021.767897

**Published:** 2021-11-02

**Authors:** Winston Koh, Shawn Hoon

**Affiliations:** Molecular Engineering Laboratory, Institute of Molecular and Cell Biology, Agency for Science, Technology and Research, Singapore, Singapore

**Keywords:** single cell RNA seq, neural network, machine learning, deep metric learning, Siamese architecture

## Abstract

Large collections of annotated single-cell RNA sequencing (scRNA-seq) experiments are being generated across different organs, conditions and organisms on different platforms. The diversity, volume and complexity of this aggregated data requires new analysis techniques to extract actionable knowledge. Fundamental to most analysis are key abilities such as: identification of similar cells across different experiments and transferring annotations from an annotated dataset to an unannotated one. There have been many strategies explored in achieving these goals, and they focuses primarily on aligning and re-clustering datasets of interest. In this work, we are interested in exploring the applicability of deep metric learning methods as a form of distance function to capture similarity between cells and facilitate the transfer of cell type annotation for similar cells across different experiments. Toward this aim, we developed MapCell, a few-shot training approach using Siamese Neural Networks (SNNs) to learn a generalizable distance metric that can differentiate between single cell types. Requiring only a small training set, we demonstrated that SNN derived distance metric can perform accurate transfer of annotation across different scRNA-seq platforms, batches, species and also aid in flagging novel cell types.

## Introduction

The field of single cell analysis has evolved rapidly over the last few years primarily driven by the development of single cell RNA sequencing (scRNA-seq) which has led to community efforts like the Human Cell Atlas (Regev et al., 2017) to enable a better appreciation of heterogeneity in complex tissues. This is paving the way for a better understanding of normal and pathological developmental programs. Many community tools have been developed that categorize heterogeneous populations of cells, based on their gene expression, into types and states (Kiselev et al., 2018; Aran et al., 2019; Barkas et al., 2019; Deng et al., 2019; Tan and Cahan, 2019; Zhang et al., 2019). Much of the effort conducted by these studies, involves careful clustering of cells and using reference markers to annotate cell types and states. This is often a time-consuming process and the reliance on a clustering process can be subjective (Aran et al., 2019). A neural network approach could address these challenges but standard deep learning techniques require large numbers of training examples to develop robust models. It is often not possible to obtain sufficient training examples to learn models for rare cell-types or disease cell states.

In this work, we are interested in exploring deep metric learning methods to train models that map cells into an embedded space where distances in this space preserves cell-cell similarity. Unlike a cell type classification objective, deep metric learning, seek to not only maximize inter cell type distance but also to minimize intra cell type distance and in so doing achieve a precise function for capturing the similarities/dissimilarities between two cells. Toward this aim, we developed MapCell, a deep metric learning based method for classifying cell types at the single-cell level by identifying similar cells, transfer annotation from labeled cell types and also facilitate the discovery of previous unseen cell types. We employed few-shot learning with a Siamese Neural Network (SNN) architecture, to learn a model that differentiate between pairs of cells using their gene expression profiles as input. Few-shot learning is a classification task where one or very few examples of each class is used to train a model to make predictions of many unknown examples. SNNs is a popular architecture that has been developed for this task because it benefits from joint learning of both a feature representation space and a distance metric, requiring few training examples to generate robust models. Siamese networks have been used in areas like signature verification (Bromley et al., 1993), image recognition (Koch et al., 2015) and facial recognition (Taigman et al., 2014), where the number of training examples for each individual class is limited and the number of classes is dynamically changing. This makes data collection and retraining costly. We find an analogous challenge in distinguishing cell types and states which can exist along a continuum and finding sufficient training examples for each state is difficult for standard architectures.

To demonstrate the use of SNN for single cell analysis, we focused on a comprehensively labeled dataset which cataloged single cell data of myeloid cells originating from matched peripheral blood and tumors of seven non-small-cell lung cancer (NSCLC) patients (Zilionis et al., 2019). We trained the SNN using 30 training examples per cell type. The process of training, deployment and validation of SNN distance metric on scRNA-seq expression data generates a reduced dimension embedding space that we used to visualize the similarities between cells. We observed that cells from types which are not represented in the training data result in consistently large distances during when compared pairwise with cell types represented in the training data, a signature which we subsequently explored for novel cell type detection. We also showed that the learned distance metric is generalizable. This is reflected when cells from cell types not represented in the training set can be distinguished from each other by projecting into the embedding space. Further refinement of the model can thus be performed by adding new reference cell-types into the embedding space without additional re-training of the model. We also demonstrate the ease of training new models by training embedding spaces for each patient in the dataset. The patient specific embedding space serves as a form of a digital twin that captures the personalized information of cell types or states. When using different patient derived models to annotate cell from a single reference patient, these patient derived models were consistent in annotating common cell types and differences only arise when particular cell types are missing from the patient specific models.

Deep metric learning methods can also scale beyond the number of cell types present in a single tissue and aid in the transfer of annotation from large scale reference atlases. We showed that a model derived from Human Cell Landscape (HCL) dataset (Guo et al., 2018) which consists of a wider survey of 843 cell types from 60 human tissue types was consistent in annotating cell types of peripheral blood when compared to a model trained primarily on peripheral blood cell data. Lastly, we demonstrated the generalizability of the cell type annotation process across different species (mouse vs. human). By using orthologous genes between mouse and human as the features, it is possible to annotate cell types of single cell mouse data using a model trained from human data.

## Results

### A Siamese Neural Network Architecture for Single Cell Gene Expression

**FIGURE 1:**
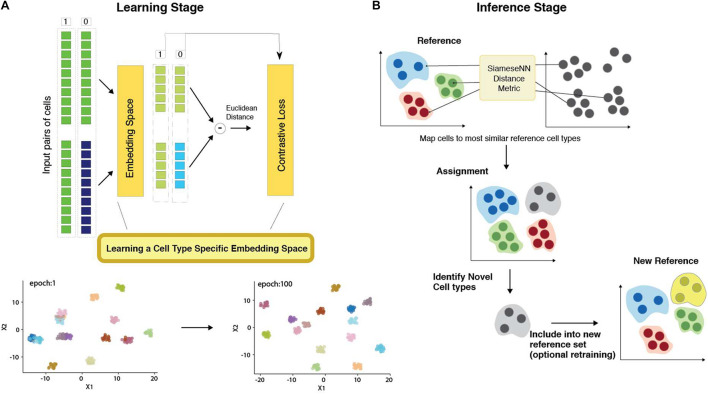
Architecture of MapCell Siamese Neural Network (SNN). **(A)** (Top) SNN architecture (Bottom) Low-dimensional representation of embedding space. **(B)** SNN inference: Each cell in the sample set is compared using the SNN metric to a set of reference cells used in the learning stage. The assignment is made to the closest reference type. Cells that do not meet the threshold are flagged as novel cell types. These novel types can be reincorporated into the training set to generate a new SNN or included in the reference set without training.

The network architecture employed in this study is illustrated in Figure 1. Our SNN consists of two identical subnetworks with shared weights. This subnetwork consists of a 3-layer neural networks with 512, 512, and 32 nodes, respectively. Dropout layers are introduced between layers to improve the generalizability of the embedding space.

To prepare the inputs for training, the counts of the most highly expressed gene is used to scale all other genes to ensure that input values are scaled between [0, 1]. Pairs of cells across cell types were fed into one of two identical subnetworks and optimization was performed using a contrastive loss function. The training process can be visualized by examining the output of the last layer composed of 32 neurons using heatmap and UMAP dimension reduction visualization (Supplementary Figure 1). The NSCLC (Zilionis et al., 2019) training set contains the same cell types originating from different tissues: peripheral blood and tumor. In the initial training epochs, cells from different cell types are already differentiated in the final neural net layer. Similar cell types found in different tissues were resolved as training further progressed. For example, B-cells from peripheral blood and tumor, were clustered together in epoch 1 but subsequently resolved in epoch 9 (Supplementary Figure 1A). Similarly, in the embedding space, tumor NK and T cell were more similar to each other than their peripheral counterparts in epoch 9 but subsequently resolved by epoch 100 (Supplementary Figure 1A). We can also observe the firing patterns of the neural network using a heatmap representation (Supplementary Figure 1B). We see that firing patterns become more discrete as training progresses. The heatmap also reflects the complexity of the learned neural network. The number of unused nodes (blue squares in Supplementary Figure 1B) suggests a less complex network could be employed for further performance optimization.

### Employing MapCell for Cell-Type Annotation

To illustrate the generalizability of using SNN distance, we used the aforementioned model trained on the inDrop scRNA-SEQ platform (Klein et al., 2015) to annotate a PBMC10K dataset generated by the 10X Chromium system, a different scRNA-seq droplet platform (10X Genomics). The 10X Chromium dataset included simultaneous cell surface protein measurements using oligonucleotide-tagged antibodies that provide an orthogonal validation of cell identity.

**FIGURE 2:**
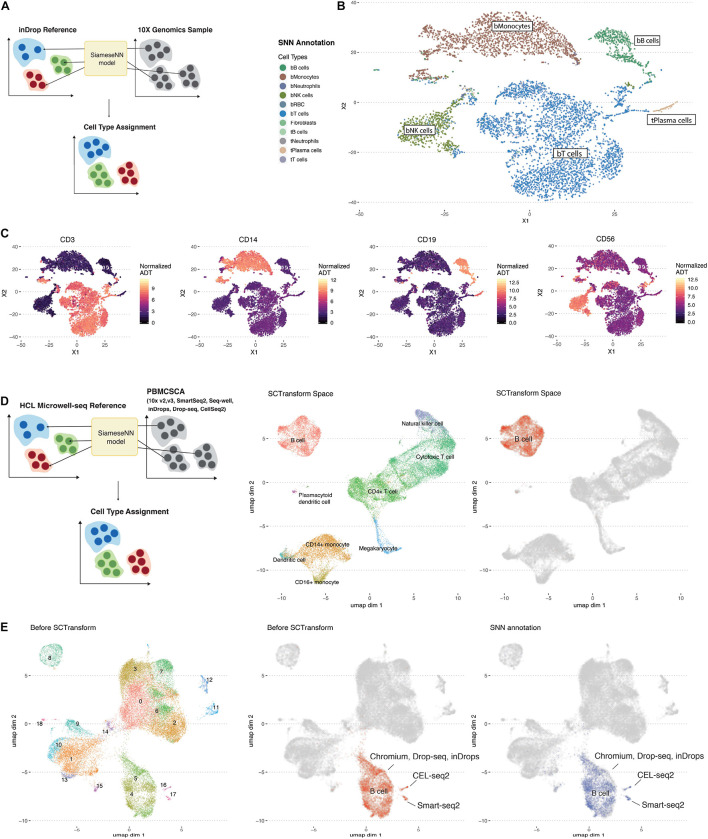
Workflow of the annotation of cell types using MapCell. **(A)** Each cell to be annotated (10X Genomics sample) is compared to 20 cells from each reference cell type using the learned MapCell model. The reference cell type with the closest average distance is assigned to the cell. **(B)** t-SNE plot of Siamese Neural Network (SNN) annotated scRNA-seq data from 10X genomics. **(C)** Cell surface protein expression mapped onto the respective cells in the same t-SNE space. Protein markers exhibit similar boundaries as the predicted annotations by SNN model. **(D)** (Left) Annotating a set of collated PBMC single-cell RNA data generated from with seven different methods using a SNN model trained on the Microwell-seq HCL PBMC dataset. (Middle) UMAP of combined data after SCTransform. Annotation was provided by Seurat. (Right) UMAP highlighting B Cell cluster after SCTransform. **(E)** (Left) UMAP of combined data before SCTransform. (Middle) UMAP highlighting B cell annotated cells before SCTransform. Indicated are clusters representing platform-specific batch effects (Right) UMAP highlighting SNN annotation of the B cell clusters before SCTransform.

The MapCell inference process compares each cell in the PBMC10K evaluation set to 20 reference cells used in the training set. The cell type with the closest average distance is assigned (Figure 2A). Five major cell-types labels (bT cells, bB cells, bMonocytes, bNK, and tPlasma cells) from the reference dataset were mapped onto the evaluation data (Figure 2B). The corresponding annotated cells clusters exhibited the canonical cell surface makers as illustrated by overlaying protein expression levels onto cells in the RNA defined t-SNE space (Figure 2C). The protein boundaries between cell clusters agree with the cell type boundaries annotated by the MapCell. Notably, for T Cells, B Cells, Monocytes and NK cells, the PBMC10K cells were mapped to the corresponding blood-derived cell types rather than the tumor-derived cell types. All plasma cells were mapped to tumor-derived plasma cells because the reference contained only this source of plasma cells.

Next we trained a model based on PBMC data generated by the HCL (Guo et al., 2018) using a Microwell-seq platform to annotate a set of PBMC data generated on seven different scRNA-seq platforms (Ding et al., 2019; Figure 2D). First the data was processed using the SCTransform batch correction function in Seurat (Stuart et al., 2019). For illustration, we highlighted the B cell cluster after batch correction (Figure 2D). Notably, platform-specific B cell clusters were observed before batch correction (Figure 2E). Despite this, we found that MapCell, which takes scaled raw cell counts as input, was batch invariant and able to identify B cells across different scRNA-seq platforms. On a desktop with a GPU, MapCell takes ∼30 s to annotate 10,000 cells (Supplementary Figure 2).

### Siamese Neural Network Distance Is a Better Contrastive Distance Metric Than Cosine and Euclidean Distances

**FIGURE 3:**
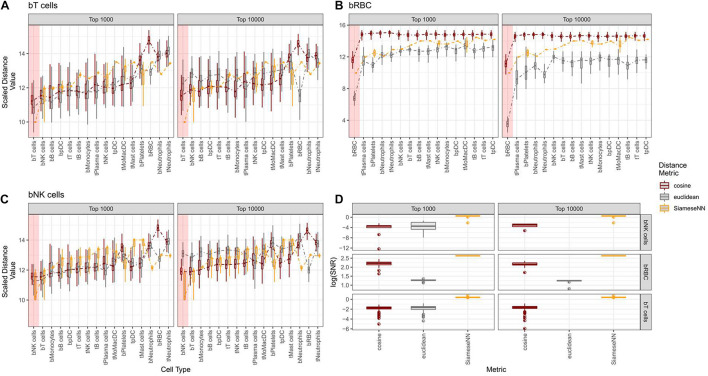
Contrasting Cosine and Euclidean against Siamese Neural Network (SNN) distance for T-cells, RBC, and NK cells. **(A–C)** Boxplots showing distribution of pairwise distance scores between each annotated cell type from the test set against annotated cell type from the training set. For cosine and euclidean distances, the top 1,000 and 10,000 most variable genes were used to calculate pairwise distance scores. For SNN, all genes were used as input to neural network. The x-axis is ordered from left to right by the SNN distance values in ascending order. Colored red box indicates the reference cell type with the average closest distance. **(D)** Boxplot showing the distibution of signal-to-noise values for each predicted cell type.

We contrasted the SNN distance metric against commonly used Euclidean and cosine distance metrics using the NSCLC (Zilionis et al., 2019) model. Twenty cells from each independently annotated cell type were compared pairwise against twenty cells across other cell types (Figures 3A–C). For cosine and Euclidean distance metrics, we picked the top 1,000 and 10,000 most variable genes while for SNN, all genes were used. We evaluate SNN’s ability to resolve cell types by the average distance between pairs of identical cell types and pairs of dissimilar cell types. We found that SNN distances for similar cell pairs were much smaller than the next dissimilar cell pair. This drop off is consistently observed for SNN distance metric across cell types. We quantified this using a signal-to-noise statistic (Figure 3D). The gain in signal is most pronounced when comparing red blood cells (RBC) across all other cell types (Figures 3B,D). As RBCs are biologically distinct from the other white blood cell types, we see a much smaller distance for RBC-RBC comparisons in contrast to other cell types. This is also reflected in the lower signal-to-noise ratio for similar cell types. This is especially evident for T and NK cells. While cosine and Euclidean distances were unable to unambiguously distinguish between T and NK cells types, SNN defined a clear demarcation between the two cell types while still ranking them as the two closest cell types (Figures 3A,D). We also found that for Euclidean and cosine distance metrics, the number of variable genes pick can impact cell type identification. For example, when 10,000 genes were used, the Euclidean distance metric failed to distinguish bNK from bT cells. This demonstrates the advantages of SNN where careful feature selection is unnecessary for optimal performance. This is important for use cases where cell types or states present may have different number of expressed genes. We also found that this feature of SNN was useful for distinguishing different cell states. It is known that lymphocytes that infiltrate the tumor have a distinct cell state from lymphocytes found in peripheral blood (Gentles et al., 2015). With both Euclidean and Cosine distance, tumor B-cells (tB cells) were not well-distinguished from peripheral B cells (bB cells) while SNN distance clearly distinguished tB cells from bB cells (Supplementary Figure 3). Taken together, we have shown that SNN distance is a robust metric for both cell type and cell state comparisons.

### Identifying Novel Cell Types

**FIGURE 4:**
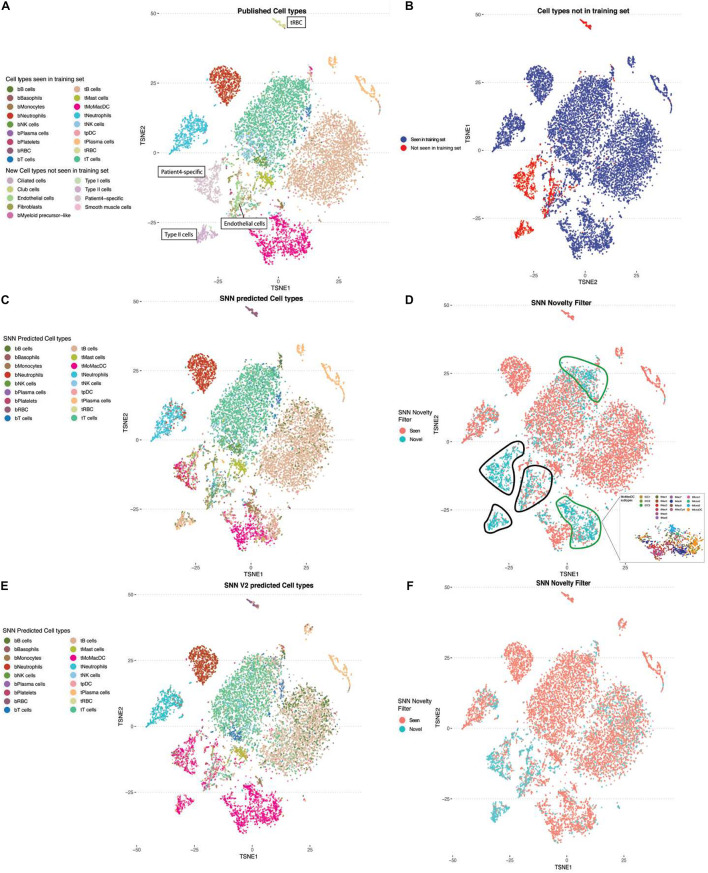
Using Siamese Neural Network (SNN) distance for novelty detection. **(A)** TSNE plot of single-cell RNA-seq NSCLC sample colored by cell types. Cell types not present in the training data set are labeled in boxes (tRBC, patient-4 specific cells, endothelial cells, Type II cells). **(B)** TSNE plot of the same data. Highlighted in blue and red are the cells which were classified as cell types found and not found in the training set, respectively. **(C)** TSNE plot colored by SNN predicted cell types. **(D)** TSNE plot colored by SNN novelty filter. Cells marked by black outline are flagged as novel cells not seen in training set. Cells marked by green lines are flagged as novel cells seen in training set. Callout box are the MoMacDC cluster colored by annotated subtypes. **(E)** TSNE plot colored by cell-type classification after re-training to include different sub-celltypes from MoMacDC cluster. **(F)** TSNE plot color by SNN novelty filter after re-training with sub-celltype MoMacDC examples.

As larger surveys of single-cell experiments are performed, we need to account for cell types and states that are not present in the training data set. For the purpose of refining the annotations and MapCell model, it is more desirable to flag these novel cells rather than assign them to the closest cell type found in the training set. We examined whether the SNN distance metric can be used in novelty detection. We selected cells from a patient with three cell types (Type II cells, endothelial cells, and patient-4 specific cells) that were not present in the training set and compared them against the reference cell types in the training data (Figures 4A,B). Predicted cell types were largely in agreement with human annotations (Figure 4C). We defined a novelty filter that will flag a cell as novel when the minimum distance computed across all cells is <2 standard deviation from the rest of comparisons. We found five regions that contained a high number of novel cell types. Expectedly, three of the five regions contained cell types not seen in the training set (black boundary, Figure 4D). The other two regions were found in the MoMacDC and T-cell clusters (green boundary, Figure 4D). Upon closer examination, we found that the MoMacDC cluster was comprised of clusters of subtypes (Zilionis et al., 2019) that were under-represented in the training set. As a result, the network did not recognize these cells as belonging to the MoMacDC cluster. We trained a new network that used the subtype labels to generate additional pairs of cells from these subtypes for training. This resulted in a more comprehensive training set and a better classification result (Figure 4E). The MoMacDC and T-cell clusters were no longer flagged as novel while the unseen training examples remained flagged as novel (Figure 4F). We used an alluvial plot to visualize the change in mapping of cell annotations after subtype training (Supplmentary Figure 4A). In agreement with the UMAP visualization, we see that after training on the new training set, we find a better mapping of the tMoMacDCs and tT cells (Supplementary Figure 4B). This demonstrates a process where a novel cell type can be automatically flagged by the MapCell for human inspection. This cell type can then be incorporated into the reference database for futher training. We did also observe, however, that a minority of the tTcells which were classified correctly before, were misannotated to a different cell type. This could reflect the quality of the underlying published sub cell type annotation or insufficient sampling of training examples from the subtypes that led to overfitting of the model.

### Siamese Network Derived Embedding Space Can Distinguish Unseen Cell Types

Requiring a retraining process is a computationally intensive process. We explored whether the contrastive nature of Siamese network learns a general function that can be applied to new cell types without re-training. The intuition is that if sufficient diversity of gene expression measurements across different cell types are seen, the network would learn to weigh different sets of genes representing pathways. These would enable new cell types, which have different combinations of pathways expressed, to be compared. Since there are unique cell types to particular patient groups in the Zilionis study (Zilionis et al., 2019), we trained on one patient set (Supplementary Figure 5A) and projected cell types that the network was not trained on into the SNN-derived embedded space (Supplementary Figure 5B). We observed that the learned embedded space retains a general capacity to distinguish previously unseen cell types during training into separate clusters. To further validate the generalizability of the feature vectors in this embedding space, we generated a K nearest neighbor graph network using 20 cells from the trained cell types. We added to this graph the novel cell types that were not previously used for training and showed that distinct new cell types formed new clusters (Type II cells, Endothelial cells, Fibroblasts). In contrast, enucleated RBC from tissue or peripheral fraction were indistinguishable reflecting their biological similarity (Supplementary Figure 5C).

### Scaling MapCell From Small to Large Models

**FIGURE 5:**
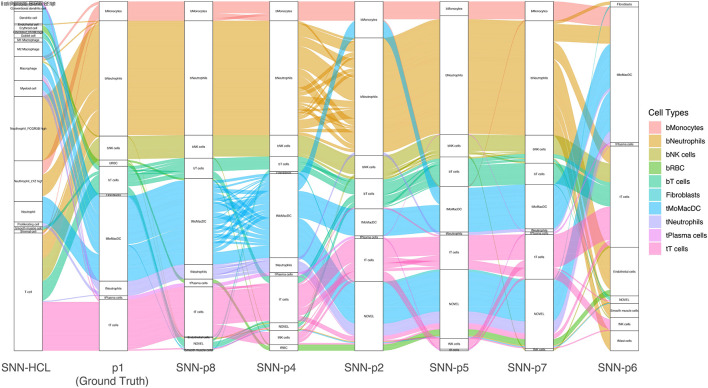
Alluvial plot of a single patient sample annotated using individually trained models derived from other patients and the Human Cell Landscape (HCL) model. Each column reflects the annotation of patient 1 data by various trained models using the SNN architecture. The leftmost column reflects annotations by Siamese Neural Network (SNN)-Human Cell Landscape model. The second column represents the original annotation for patient 1. The remaining columns represents annotations by models trained with different patient data (p2, p4, p5, p6, p7, p8). Cell Types are colored with respect to p1 (ground truth) annotations.

Each patient profiled in the lung cancer dataset (Zilionis et al., 2019) contained different numbers and diversity of cell types. To test the robustness of MapCell models, we trained a unique model for each patient, leaving one patient out for validation. We treated each individually trained model as a pseudo-digital twin of the original patient. An alluvial plot is used to visualize the consistency and differences in annotations using personalized embedding spaces (Figure 5). We compared these small personalized models against a large model developed with a generalized embedding space that is capable of contrasting a large diversity of cell types. The HCL (Guo et al., 2018) comprises a wide survey of cell types derived from about 50 different tissues. There are close to 700,000 cells in the data with 384 cell types and we sampled cells from cell types that are represented by at least 30 cells. The sampled cells were used to generate pairs of contrasting cells for training. We used this HCL model to annotate the held-out sample. This demonstrated the scalability of the MapCell architecture and its capability in accommodating cell types numbers on the order of an entire human cell atlas. Concordance of the major cell types were observed when comparing the annotations from the patient’s model as well as the HCL model. We also observed that the HCL model did not distinguish between T-cells of blood and tumor origin likely because these contrasting cell types were absent in the HCL dataset.

### Generalizing MapCell for Interspecies Annotation Transfer

**FIGURE 6:**
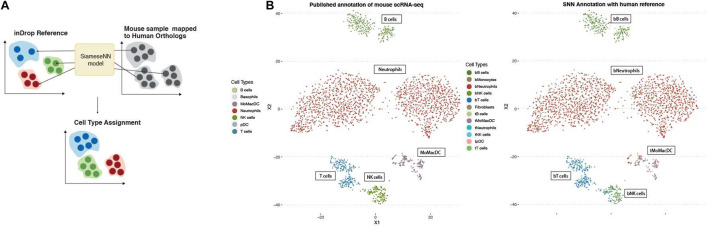
Inter-species annotation transfer. **(A)** Transfer annotation from human to mouse. Mouse genes were mapped to human orthologs and MapCell prediction was performed on mouse scRNA-seq data using the NSCLC model. **(B)** (Left) Published annotation of mouse scRNA-seq data (Right) SNN predicted cell types using human reference.

While the availability of single-cell genomics makes it possible to profile cells form different organisms, it is still a costly endeavor to generate atlases for multiple non-model organisms. Next we tested whether the MapCell can be used to transfer annotation to a related species. Mouse genes were mapped to human orthologs and MapCell prediction was performed on single-cell RNA-seq data of PBMC from a healthy mouse using a human reference. We showed that we could successfully annotate the mouse sample using the MapCell trained from human data (Figure 6).

## Discussion

We demonstrated the application of Siamese networks as a similarity function and demonstrated its usage in annotating cell types from single-cell RNASeq experiments. Training with this neural architecture requires only a small number of representative cells (30 in this study), making it ideal for learning of cell features of potentially rare cell types or transient states. Despite the small training set, we demonstrated that the MapCell can perform predictions across different scRNA-seq platforms, identify novel cell types and transfer annotations across species. Our SNN-derived distance metric is robust compared to Euclidean and cosine distance. It can serve as a generalized metric for making comparisons for cell-types not seen in the training set. This allows the inclusion of cell-types in the reference database without the need for re-training. Furthermore, the SNN distance metric can be integrated with other machine learning algorithms that employ distance metrics such as K-Nearest Neighbor (KNN) for rapid deployment. In our work, we have deployed models comparing different patients as a means to detect private cell types. It is conceivable that such an approach can be applied for a single patient comparing multiple timepoints against a baseline model. Such a baseline model can be thought of as a digital twin of the patient capturing the diversity of the patient cell types and states in the trained embedding space.

While we tried to demonstrate a breadth of possible single cell analytical scenarios possible within the Siamese framework, we recognize there is a limitation in our exploration. There remains many other similar architecture types such as the triplet network and a wide range of loss functions e.g., Quadruple Loss, Structured Loss, N-paired Loss. These other networks can also be paired with a variety of different sample selection scheme for even more efficient training. Nevertheless, we believe that our characterization with a relatively straight forward implementation of Siamese based neural network have validated the potential for greater exploration of using one-shot deep metric learning approaches toward understanding single cell sequencing data. In our future work, we foresee advances in single cell technologies that allows for simultaneous measurements of different data modality from a single cell such as protein marker expression, chromatin occupancy and DNA mutations. This diversity in single cell data results will result in novel situations and we believe deep metric learning approaches can help extract knowledge from the volume, diversity, and complexity of such datasets.

## Materials and Methods

### Siamese Network Architecture and Training

#### Architecture

The architecture of the Siamese network as its name implies has two inputs vectors **X_1_, X_2_** that feeds into a common neural network that shares the same weights ***W***. This dense fully connected neural network consists of an input layer with 33,694 nodes, each corresponding to a specific gene, followed by 2 fully connected layers each with 512 hidden nodes and a final 32 nodes output layer. The final output layer represents a 32-dimension feature space that is intended for separation of different cell types. A custom distance layer takes the transformed vectors and calculates the Euclidean distance in this embedded space:


$$D_{w}\,\left(X_{1},X_{2}\right)=\sqrt[{2}]{\sum_{i=1}^{32}{\left(X_{1}-X_{2}\right)}^{2}}$$

#### Generalizability

Between each fully connected layer, an additional dropout layer at a rate of 0.5 is implemented during training to ensure generalizability of the network during implementation. This network is implemented and trained using Keras and TensorFlow in both R and python environments.

#### Training With Contrastive Loss

Thirty cells are randomly selected from each cell type. Selected cells are split into training (20 cells per type) and validation sets (10 cells per type). Across the selected 20 cells of each types, pairs are generated: pairs originating from same cell type are labeled as 1 and pairs of cells from different cell types are labeled as 0. Gene counts of each cell are normalized by scaling with the maximum gene count of the cell. The binary cell labels **Y**, and Euclidean distance of the two-feature vector derived above **D_*w*_** is fed into the contrastive loss function:


$$L_{w}\,\left(Y,D_{w}\right)=\left(1-Y\right)\,\frac{1}{2}\,{\left(D_{w}\right)}^{2}+\frac{1}{2}\,{\left\{\max\left(0,m\,a\,r\,g\,i\,n-D_{w}\right)\right\}}^{2}$$

This loss was back-propagated to calculate the gradient and RMSprop (Hinton et al., 2012) was used to update the weights.

### Visualization of Training Process

Visualization of the training process begins with calling back the weights of the neural networks across the training epochs. Weights corresponding to each training epoch are loaded, and each cell’s gene expression vector are passed through the network, where the final output of the embedding layer of a vector length 32 for each cell are collected and reduced into a two-dimensional space using UMAP. Individual firing of each of the 32 nodes in the final layer of neural networks are also visualized using heatmaps using the R package ComplexHeatmap (Gu et al., 2016).

### Comparison of Siamese Neural Network Distance With Euclidean and Cosine Distance

Twenty cells are randomly selected from each of the annotated cell clusters of reference patient data. Each of these cells are paired with 20 other cells from the other annotated clusters. The distance between the 20 pairs of cells across the different annotated cell types are calculated using the SNN, Euclidean and cosine metric. The resulting distance for the distance metric is visualized using bar graphs in Figure 2. In order to quantitate the contrast in distance between the exact match and second-best match in terms of annotated cell types, we calculate the Signal to Noise Ratio between the top two matches:


$$S\,N\,R=\frac{\left|\mu_{1}-\mu_{2}\right|}{\left|\sigma_{1}+\sigma_{2}\right|}$$

where μ_1_ and μ_2_ represent the average distance of 20 cells for each pair of cell type, respectively, and σ_1_ and σ_2_ represent the standard deviation of the same 20 cells.

### Validation of Siamese Neural Network Distance Usage in Annotating External Datasets

PBMC3K dataset was obtained from the 10X genomics.^1^ PBMCSCA data set was obtained from the SeuratData (Stuart et al., 2019) distribution. The PBMC3K dataset contains both gene expression and cell surface protein expression data from single cells. Each cell gene expression vector is matched up accordingly to the gene inputs that the Siamese model was trained on. Each of the external single cell gene vector is then paired against the trained reference cell types and fed into the Siamese network to obtain the SNN distance. The cell type of the reference cell group that correspond to the lowest SNN distance is then used to annotate the cell.

### Validation of Siamese Neural Network Distance Usage in Annotating Different Species

Single cell gene expression data from mouse samples in the same study was mapped to orthologous human genes using Mouse Genome Informatics (MGI).^2^ Human genes with no known mouse orthologs are set to zero. This transformed input is then paired against the trained reference cell types and fed into the Siamese network to obtain the SNN distance. The cell type of the reference cell group that correspond to the lowest SNN distance is then used to annotate the cell.

### Generalizability of Siamese Trained Embedding Space in Distinguishing Cell Types

Single cell gene expression vector from cell types not used during the training of the model were selected and embedded using the prior trained embedding neural net. KNN is then performed on the feature vectors to generate a KNN graph network. Visualization of separations in the novel cell clusters within the network is achieved by using Fruchterman-Reingold force layout.

### Generation and Annotation Using Human Cell Landscape Atlas as a Reference

Raw data was obtained from the HCL portal^3^. Using the cell annotations provided, we tallied the different cell types within each tissue type. Only cell types, within each tissue, that have at least 30 cells were used for training. Twenty cells are sampled from each cell type to generate pairs for training. The remaining 10 cells are used for validation. A binary indicator vector of same length to the number pairs is also generated where 1 indicates the pair of cells are drawn from the same cell type and 0 otherwise. The prepared data of cell pairs is fed into the SNN architecture as defined earlier.

For training the HCL dataset, the computational demand on hardware memory necessitated running the training on an AWS p2.large instance. All other training runs were performed on a local desktop with a RTX-2080 GPU. Callbacks were made to save the weights of the network at each epoch. To evaluate the progress of the training, a Siamese accuracy metric defined by arbitrarily setting the Euclidean distance at 0.5 where a distance lower than 0.5 is deemed that the cells are derived from the same cluster and conversely, distances greater than that are determined to be cells from different cell cluster. Weights from the epoch that gives the highest achievable training and validation accuracy are retained for deployment during annotation phase. Using the learned embedding from the network, the dimension reduced vectors of these reference cell groups are used to generate a reference KNN network. For the annotation phase, each of single cell vector from the Zilionis dataset is projected into the same space, and annotation is transferred using K nearest neighbor with K set at 3.

### Generation of Digital Twins *via* Embedding Space of Siamese Neural Network

Using the same process of training the HCA model, the process is repeated across each of the patients in the Zilionis dataset. A different embedding space is derived from each of the patient’s trained network. Each of these embedding spaces is used to annotate the same held-out patient test dataset. Comparisons of the resulting cell type annotation from using the different embedding schemes are visualized using alluvial plots in R using ggalluvial package.

### Interspecies Annotation Using Siamese Neural Network

To use the human trained SNN model for mouse annotation, we first obtained the mouse-human orthologs from MGI (see text footnote 2). Single cell RNA-seq data from mouse with a human orthologs are mapped to the same input using the SNN. The rest of the human gene inputs with no corresponding mouse orthologs are set to zero. The resulting inputs are compared to the human reference cell with known annotations and the three nearest reference human cells in the embedded space identified by K nearest neighbor were used to annotate the mouse cell.

### Code and Application Programming Interface

Sample code and trained models described in this paper are available for download at https://github.com/lianchye/mapcell. We have also hosted the trained model on AWS and provided an application programming interface (API)^4^ that abstracts away the need for deployment for annotation. Each http GET request will send a JSON formatted single cell gene vector to the API which will annotate a single cell within 300 s, below the timeout limit (900 s) of AWS lambda functions. While this cloud deployment scheme, will be slower in deployment compared to a local server model, we believe that a cloud deployment allows for much easier access to the trained model and has the scalability to better serve the wider community.

## Data Availability Statement

The datasets presented in this study can be found in online repositories. The names of the repository/repositories and accession number(s) can be found below: https://github.com/lianchye/mapcell.

## Author Contributions

WK wrote the code for the machine learning algorithms. Both authors came up with the concept and performed the computation experimentation.

## Conflict of Interest

SH is a cofounder of Proteona Pte., Ltd. The remaining author declares that the research was conducted in the absence of any commercial or financial relationships that could be construed as a potential conflict of interest.

## Publisher’s Note

All claims expressed in this article are solely those of the authors and do not necessarily represent those of their affiliated organizations, or those of the publisher, the editors and the reviewers. Any product that may be evaluated in this article, or claim that may be made by its manufacturer, is not guaranteed or endorsed by the publisher.

## Footnotes

1
https://support.10xgenomics.com/single-cell-gene-expression/datasets/3.0.2/5k_pbmc_v3

2
http://www.informatics.jax.org/downloads/reports/HMD_HumanPhenotype.rpt

3
https://db.cngb.org/HCL/

4
http://13.229.250.159:8000/__swagger__/

## Supplementary Material

Supplementary Figure 1 Visualization of training phase of Siamese Neural Network (SNN). (Top Row) Umap visualization of the embedding space projection in the last neuronal output at different training epochs. Example cell types that are better resolved, as measured by increased spatial separation in the embedding space over increasing training epochs, are indicated with arrows. (Bottom Row) Heatmap representation of the neural network firing pattern where each row is a cell and each column a single neuron in the final output layer.

Supplementary Figure 2 Annotation performance of MapCell. Speed of MapCell annotation on a local desktop with a RTX-2080 GPU.

Supplementary Figure 3 Contrasting Cosine and Euclidean against SNN distances for distinguishing cell state. **(A)** Peripheral B cells (bB cells) and tumor derived B cells (tB cells) from the test set are compared against the reference cell types in the training set. **(B)** Boxplot showing the distibution of signal-to-noise values for the different distant metrics for blood and tumor derived B-cells.

Supplementary Figure 4 Alluvial plot depicting the switch in novelty status and annotation status when incorporating left out subtypes during training of SNN models. **(A)** Mapping of cell types based on SNN trained on major cell type selected training examples. **(B)** Mapping of cell types based on SNN trained on minor cell type selected training examples. Addition of omitted minor clusters of cell types redirects the annotations from novel to identifiable, and each to its respective expected human annotated states. The process depicts the capability of the SNN network to be used as an novelty detector as well as the plasticity of such a process to allow for subsequent update of novel classes.

Supplementary Figure 5 Siamese derived embedding space. **(A)** K-nearest neighbor (KNN) graph network of Siamese Network embedding space trained on a single patient. **(B)** Projection of cell types not trained in the initial network onto embedding space. **(C)** KNN graph network of Siamese Network embedding space with new cell types incorporated.
