# Supplementary figures and images for: MapCell: Learning a Comparative Cell Type Distance Metric With Siamese Neural Nets With Applications Toward Cell-Type Identification Across Experimental Datasets
Source: Front Cell Dev Biol. 2021 Nov 2;9:767897. doi: 10.3389/fcell.2021.767897 (PMC8593221; doi:10.3389/fcell.2021.767897)

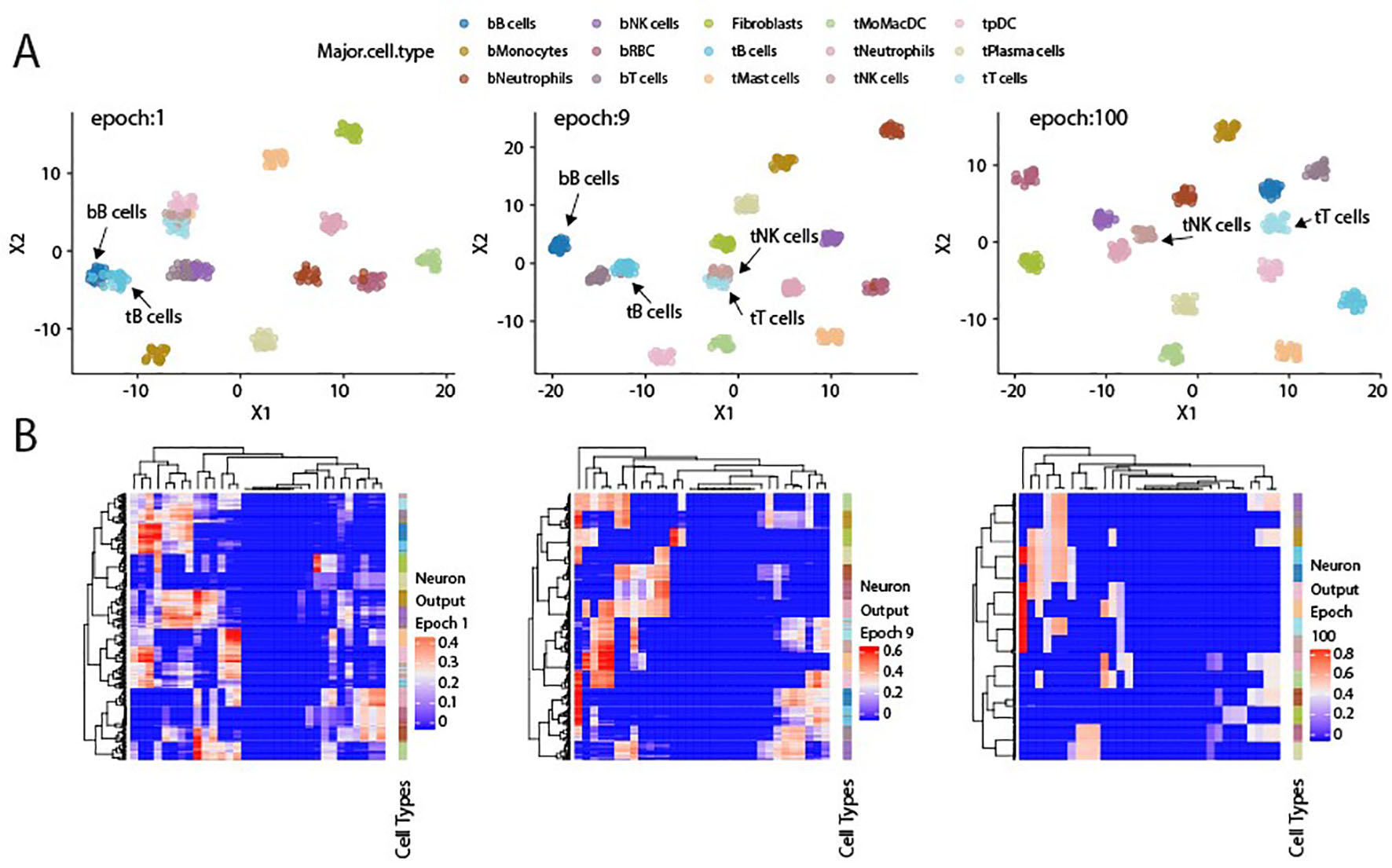

Supplement: Supplementary Figure 1 — Visualization of training phase of Siamese Neural Network (SNN). (Top Row) Umap visualization of the embedding space projection in the last neuronal output at different training epochs. Example cell types that are better resolved, as measured by increased spatial separation in the embedding space over increasing training epochs, are indicated with arrows. (Bottom Row) Heatmap representation of the neural network firing pattern where each row is a cell and each column a single neuron in the final output layer. [file Image_1.JPEG]

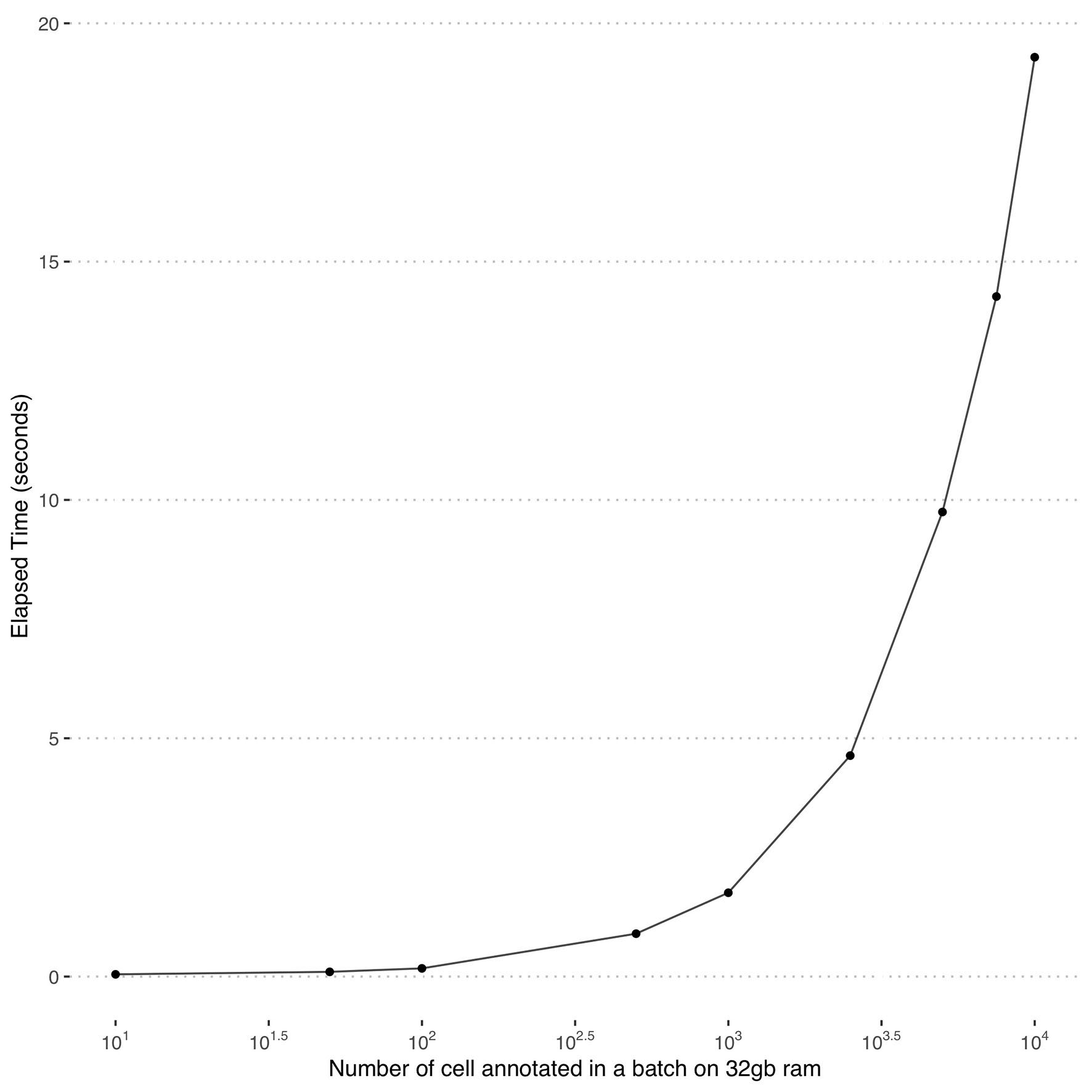

Supplement: Supplementary Figure 2 — Annotation performance of MapCell. Speed of MapCell annotation on a local desktop with a RTX-2080 GPU. [file Image_2.JPEG]

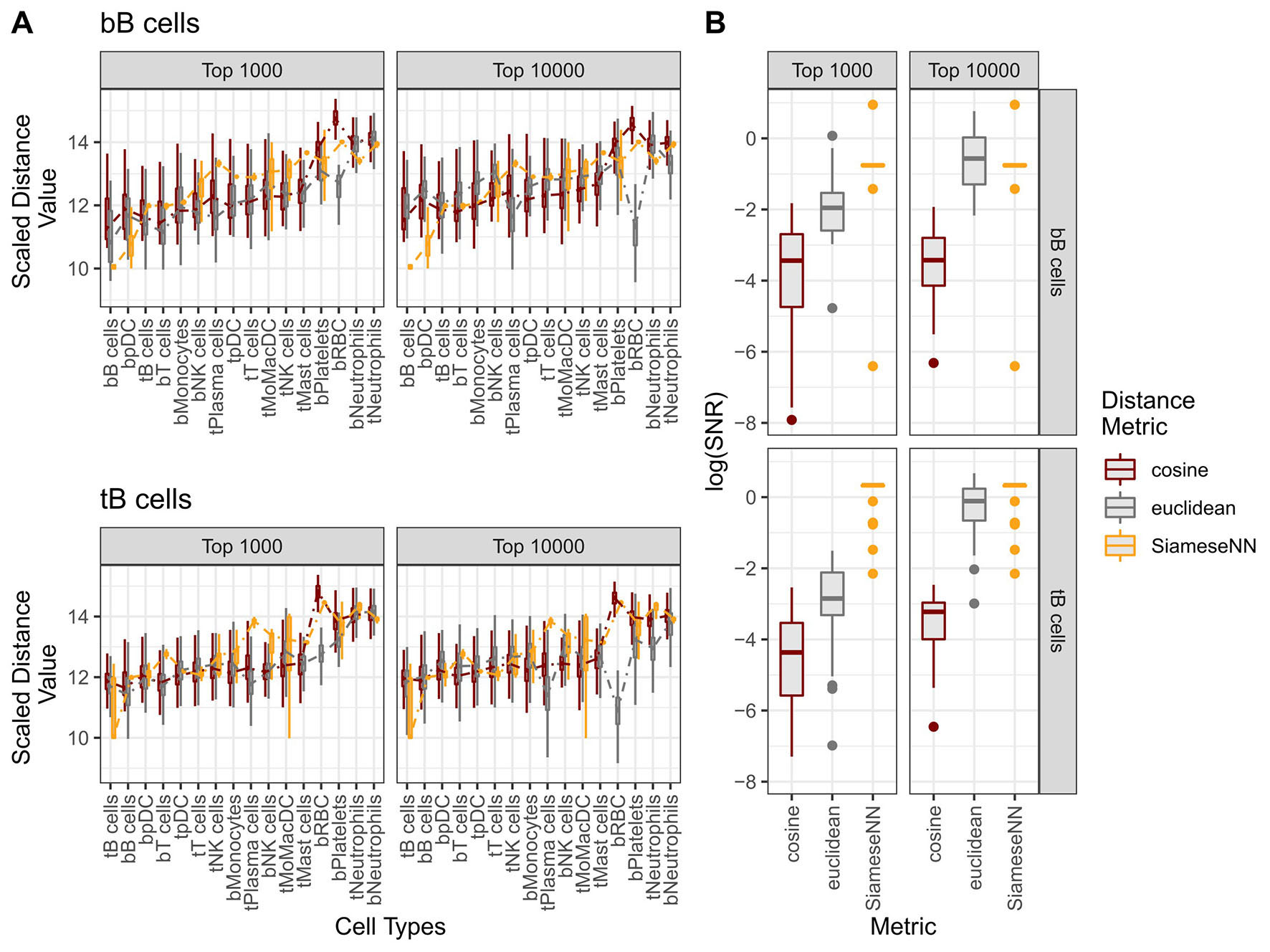

Supplement: Supplementary Figure 3 — Contrasting Cosine and Euclidean against SNN distances for distinguishing cell state. (A) Peripheral B cells (bB cells) and tumor derived B cells (tB cells) from the test set are compared against the reference cell types in the training set. (B) Boxplot showing the distibution of signal-to-noise values for the different distant metrics for blood and tumor derived B-cells. [file Image_3.JPEG]

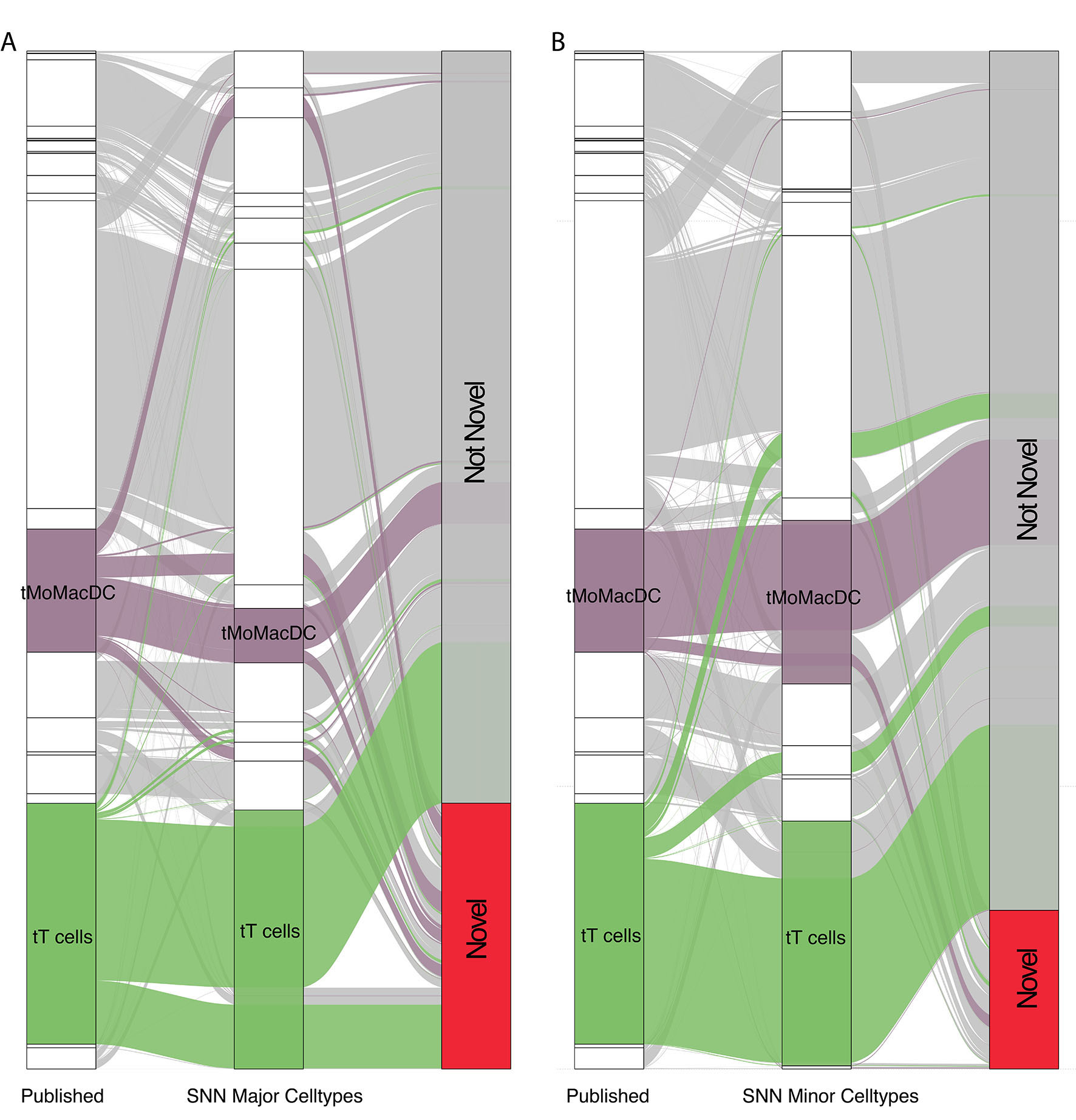

Supplement: Supplementary Figure 4 — Alluvial plot depicting the switch in novelty status and annotation status when incorporating left out subtypes during training of SNN models. (A) Mapping of cell types based on SNN trained on major cell type selected training examples. (B) Mapping of cell types based on SNN trained on minor cell type selected training examples. Addition of omitted minor clusters of cell types redirects the annotations from novel to identifiable, and each to its respective expected human annotated states. The process depicts the capability of the SNN network to be used as an novelty detector as well as the plasticity of such a process to allow for subsequent update of novel classes. [file Image_4.JPEG]

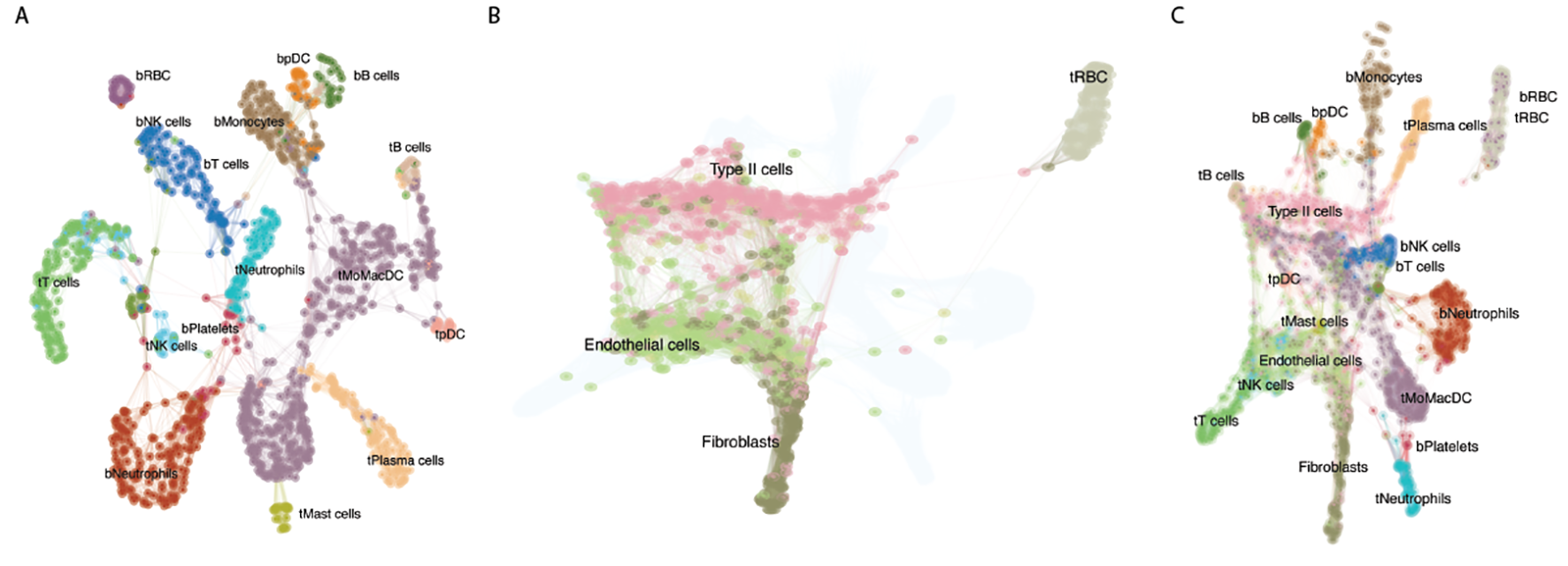

Supplement: Supplementary Figure 5 — Siamese derived embedding space. (A) K-nearest neighbor (KNN) graph network of Siamese Network embedding space trained on a single patient. (B) Projection of cell types not trained in the initial network onto embedding space. (C) KNN graph network of Siamese Network embedding space with new cell types incorporated. [file Image_5.TIF]
